# Preemption versus Entrenchment: Towards a Construction-General Solution to the Problem of the Retreat from Verb Argument Structure Overgeneralization

**DOI:** 10.1371/journal.pone.0123723

**Published:** 2015-04-28

**Authors:** Ben Ambridge, Amy Bidgood, Katherine E. Twomey, Julian M. Pine, Caroline F. Rowland, Daniel Freudenthal

**Affiliations:** 1 University of Liverpool, Liverpool, United Kingdom; 2 University of Lancaster, Lancaster, United Kingdom; 3 ESRC International Centre for Language and Communicative Development (LuCiD), Manchester, Liverpool and Lancaster, United Kingdom; University of Texas Health Science Center at San Antonio, Research Imaging Institute, UNITED STATES

## Abstract

Participants aged 5;2-6;8, 9;2-10;6 and 18;1-22;2 (72 at each age) rated verb argument structure overgeneralization errors (e.g., *Daddy giggled the baby) using a five-point scale. The study was designed to investigate the feasibility of two proposed construction-general solutions to the question of how children retreat from, or avoid, such errors. No support was found for the prediction of the *preemption* hypothesis that the greater the frequency of the verb in the single most nearly synonymous construction (for this example, the periphrastic causative; e.g., *Daddy made the baby giggle*), the lower the acceptability of the error. Support was found, however, for the prediction of the *entrenchment* hypothesis that the greater the *overall* frequency of the verb, regardless of construction, the lower the acceptability of the error, at least for the two older groups. Thus while entrenchment appears to be a robust solution to the problem of the retreat from error, and one that generalizes across different error types, we did not find evidence that this is the case for preemption. The implication is that the solution to the retreat from error lies not with specialized mechanisms, but rather in a probabilistic process of construction competition.

## Introduction

The story of language acquisition is, in large part, the story of how children move beyond simply repeating words and phrases that they have learned from their caregivers (e.g., *Bye+bye*; *Drink*!) and acquire the ability to produce utterances that they have never encountered in exactly that form; a point perhaps most famously made in Chomsky’s [1] review of Skinner’s *Verbal Behavior*. [2]

At the heart of this ability lies what many researchers have referred to as a paradox ([3], [4], [5], [6], [7], [8]) In order to achieve adult-like productivity with language, children must set up generalizations that allow them to use verbs in unattested constructions. For example, on the basis of hearing pairs of utterances like *The ball rolled* and *The man rolled the ball*, the child might set up a rule that allows any verb attested in the *intransitive inchoative* construction to be generalized into the *transitive causative* construction, and vice versa. Thus, upon encountering an utterance like *The vase broke*, the child could use her generalization to produce *Daddy broke the vase*, even if she has never encountered *break* in the transitive causative construction. The paradox arises because children must learn that, of the verbs that are as yet unattested in the target construction, many can be generalized into that construction if the communicative need arises (e.g., *The car moved → Mummy moved the car*), while others cannot (e.g., *The baby giggled* → *Daddy giggled the baby).

The problem of how children learn to avoid these overgeneralizations of verb argument structure is sometimes referred to as the problem of the *retreat from overgeneralization*. Indeed, findings from several naturalistic and experimental studies suggest that at least some children pass through a stage in which they produce such errors before subsequently retreating from them (eg, [7], [8], [9], [10], [11], [12], [13]) Some examples of these errors are given in Table 1. However, it is important to note that the paradox of partial productivity applies whether or not a particular child happens to produce errors from which to “retreat”. Thus, while children certainly appear to make some use of corrective feedback from adults (eg, [14]), this is unlikely to be a complete solution.

**Table 1:** Possible and attested verb argument structure overgeneralization errors.

	**Causative alternation**	
	(a) Intransitive	(b) Transitive
Alternating	The ball rolled	The man rolled the ball
(a) only	The man laughed Do you want to see our heads disappear? I don't want any more grapes; I'll cough I (didn't) giggle(d) Will I climb up there? Did it bleed? I always sweat [when I wear it] [They're nice enough that] I wish I had one	*The clown laughed the man *Do you want to see us disappear our heads? (6;0) *I don't want any more grapes; they just cough me (2;8) *Don't giggle me (3;0) *Will you climb me up there (3;2) *Did she bleed it? (3;6) *It always sweats me *[They're nice] enough to wish me that I had one (5;8)
(b) only	*I better put it down there so that it won’t lose (3;7) *They don’t seem to see Where are they? (3;8) *Do you think it’ll fix? (8;3)	I’d better put it down there so that I won’t lose it I can’t seem to see them Where are they? Do you think you can fix it?
	**Dative alternation**	
	**(a) Prepositional-object (PO) dative**	**(b) Double-object (DO dative)**
Alternating	The boy gave a present to the girl	The boy gave the girl a present
(a) only	The boy dragged the box to the girl The boy suggested the trip to the girl I said no to her Shall I whisper something to you?	*The boy dragged the girl the box *The boy suggested the girl the trip *I said her no (3;1) *Shall I whisper you something? (7;8)
	**Locative alternation**	
	**Contents (figure) locative**	**Container (ground) locative**
Alternating	The boy sprayed paint onto the statue	The boy sprayed the statue with paint
(a) only	The boy poured water into the cup Mommy, I poured water onto you I don't want it because I spilled orange juice onto it	*The boy poured the cup with water *Mommy, I poured you [M: You poured me?] Yeah, with water (2;11) *I don't want it because I spilled it of orange juice (4;11)
(b) only	*The boy filled water into the cup *I'm gonna cover a screen over me (4;5) *Can I fill some salt into the bear [-shaped salt shaker]? (5;0)	The boy filled the cup with water I'm going to cover myself with a screen Can I fill the bear with salt?
	**Passive alternation**	
	(a) active transitive	(b) passive transitive
Alternating	The girl kicked the boy	The boy was kicked by the girl
(a) only	The book cost £5	*£5 was cost by the book

Attested errors (from [7]) are shown in **bold**, with the age of the child (years;months) and a possible grammatical formulation using the alternate construction

The present study investigates two solutions to this problem It is important to also acknowledge the existence of a third, and potentially complementary, proposal: Pinker’s [8] semantic verb class hypothesis, which holds that learners form classes of verbs that are semantically (in)consistent with particular constructions For example, verbs of semi-voluntary emotional expression may appear in the intransitive and periphrastic causative constructions (eg, *The baby laughed/giggled; Daddy made the baby laugh/giggle*), but not the transitive causative (eg, *Daddy laughed/giggled the baby) The present study does not investigate this hypothesis, and we will say no more about it here, other than to note that an effect of verb semantics—though not necessarily of discrete semantic classes per se—has been observed in a number of previous studies (eg, [10], [12], [15], [16], [17], [18], [19], [20], [21], [22])

The two proposals that are investigated in the present study are both forms of statistical learning The first is the *preemption* hypothesis:

If a potential innovative expression would be precisely synonymous with a well-established expression, the innovation is normally pre-empted by the well established term, and is therefore considered ungrammatical ([5]: 798)

Preemption was first proposed to account for the retreat from word-level errors involving derivational morphology. For example, children’s novel coinages such as *spyer, *driller and *unappear are gradually preempted by the adult forms *spy*, *drill* and *disappear* (perhaps even semi-explicitly, as the child “notices” a mismatch between the adult form and her own). Because preemption is a probabilistic process, the prediction that follows from this proposal is that the greater the frequency of the competitor form, the less likely children will be to produce the error, and the more unacceptable they will rate this error in a judgment task. Indeed, recent studies of overgeneralizations of derivational morphology have provided support for this claim (see [12], [20], for errors of verbal *un-*prefixation—e.g., *unclose—and [23] for errors of adjectival *a-*prefixation—e.g., *The asleep boy).

While *preemption* works well for word-level morphological overgeneralizations, attempts to apply the proposal to utterance-level syntactic overgeneralizations (eg, [6], [24])—the focus of the present study—have met with mixed success. Again, the prediction is of a negative correlation between the production probability/rated acceptability of a particular error (e.g., *Daddy laughed the baby; a transitive causative overgeneralization) and the frequency of this verb in the single most nearly synonymous construction (e.g., *Daddy made the baby laugh*; the periphrastic causative construction). Similarly, overgeneralizations into the intransitive construction (e.g., *The book lost*) are held to be probabilistically preempted by passive uses (e.g., *The book was lost*); See Table 1 for more examples.

Although novel verb laboratory studies of the intransitive and transitive constructions have provided support for this prediction ([10], [11]) there is reason to doubt that the relevant preempting constructions are sufficiently frequent in the input to which children are exposed. Indeed, on the basis of the counts obtained for the present study, periphrastic causative and passive uses are extremely rare in child directed speech (0.4% and 2.2%). Neither does preemption seem likely to constitute a plausible mechanism by which children could avoid errors with the passive construction (e.g., *An hour was lasted by the film; A kilogram was weighed by the box; Marge was looked like/resembled by Lisa). These verbs are not particularly frequent in the potentially-preempting active construction (e.g., *The film lasted an hour*) and indeed, are presumably less frequent in this construction than many verbs that passivize easily (e.g., *Homer was pushed by Marge*). Potentially more straightforward are the three-argument constructions: datives and locatives, for which—in each case—the two alternate syntactic forms seem to express almost identical meanings (see Table 2), and hence are good candidates for preemption. Indeed, a grammaticality judgment study of errors involving the DO-dative construction (e.g., *I said her no) [21] observed a clear effect of preemption (e.g., *I said no to her*), even after controlling for another statistical predictor; entrenchment (discussed below) On the other hand, a similar study with errors involving the locative constructions (e.g., *The boy poured the cup with water; *The boy filled water into the cup) found an effect of preempting alternatives (e.g., *The boy poured water into the cup; The boy filled the cup with water*), but one that disappeared after controlling for entrenchment [18]

**Table 2:** Verbs and rated sentences.

Alternation	Verb Type	Construction	Grammatical	Sentence Pair A (high/low frequency verb)	Sentence Pair B (high/low frequency verb)	Preempting construction: *example*
**Causative** Ambridge et al (2008, Cognition) Ambridge et al (2011, Cog Ling) Bidgood et al (in preparation)	Intransitive-only	Intransitive	Yes	The cup fell/tumbled	The girl laughed/giggled	NA
		Transitive	No	*Lisa fell/tumbled the cup	*Bart laughed/giggled the girl	Periphrastic: *John made Bill laugh/giggle*
		Periphrastic	Yes	Lisa made the cup fall/tumble	Bart made the girl laugh/giggle	NA
	Transitive-only	Intransitive	No	*The ball hit/struck	*The money took/removed	Passive: *Bill was hit/struck (by John)*
		Transitive	Yes	Marge hit/struck the ball	Marge took/removed the money	NA
		Periphrastic	No	*Marge made the ball hit/strike	*Marge made the money take/remove	Transitive: *Bill hit/struck John*
	Alternating	Intransitive	Yes	The plate broke/smashed	The toy moved/rolled	NA
		Transitive	Yes	Homer broke/smashed the plate	Marge moved/rolled the toy	NA
		Periphrastic	Yes	Homer made the plate break/smash	Marge made the toy move/roll	NA
**Dative** Ambridge et al (2012, Language)	PO-only	PO	Yes	Bart carried/hauled the box to Lisa	Marge screamed/shrieked the warning to Homer	NA
		DO	No	*Bart carried/hauled Lisa the box	*Marge screamed/shrieked Homer the warning	PO: *John carried/hauled the package to Bill*
	DO-only	PO	No	*Bart cost/fined £50 to Marge	*Marge refused/denied the beer to Homer	DO: *John cost/fined Bill £10*
		DO	Yes	Bart cost/fine Marge £50	Marge refused/denied Homer the beer	NA
	Alternating	PO	Yes	Lisa gave/handed the book to Bart	Lisa showed/taught the answer to Homer	NA
		DO	Yes	Lisa gave/handed Bart the book	Lisa showed/taught Homer the answer	NA
**Locative** Ambridge et al (2011, Cognition) Bidgood et al (2004, PLOS ONE)	Figure-only	Figure	Yes	Homer poured/dripped water into the cup	Marge spilt/dribbled juice onto the rug	NA
		Ground	No	*Homer poured/dripped the cup with water	*Marge spilt/dribbled the rug with juice	Figure loc: *John poured / dribbled beer onto Bill*
	Ground-only	Figure	No	*Lisa filled/lined paper into the box	*Bart covered/coated mud onto Lisa	Ground loc: *John filled/lined the container with cardboard*
		Ground	Yes	Lisa filled/lined the box with paper	Bart covered/coated Lisa with mud	NA
	Alternating	Figure	Yes	Lisa sprayed/sprinkled water onto the roses	Homer splashed/spattered water onto Marge	NA
		Ground	Yes	Lisa sprayed/sprinkled the roses with water	Homer splashed/spattered Marge with water	NA
**Passive** Ambridge et al (submitted)	Active-only	Active	Yes	The film lasted an hour/The box weighed a kilogram	Lisa looked like/resembled Marge	NA
		Passive	No	*An hour was lasted by the film/A kilogram	*Marge was looked like/resembled by Lisa	Active: *John looked like/resembled Bill*
				was wighed by the box		
	Passive only	Active	NA	NA	NA	NA
		Passive	NA	NA	NA	NA
	Alternating	Active	Yes	Homer kicked/patted Marge	Marge pushed/chased Homer	NA
		Passive	Yes	Marge was kicked/patted by Homer	Homer was pushed/chased by Marge	NA

Thus the status of preemption as a construction-general solution to the paradox of partial productivity is unclear. While it seems to work well for morphological overgeneralizations and those involving the dative constructions, this is not necessarily the case for other argument structure constructions. In the present study we investigate whether preemption operates across a range of argument structure constructions, using statistical techniques that allow us to generalize beyond the particular constructions tested: mixed effects models ([25], [26])

A more recent statistical learning proposal—and one that perhaps does not share the intuitive appeal of preemption—is *entrenchment* [27]. The general idea goes back to Langacker [28] who posits a continuous scale of entrenchment in cognitive organization. Every use of a structure has a positive impact on its degree of entrenchment. Units are variably entrenched depending on the frequency of their occurrence.

When applied to the domain of the retreat from overgeneralization (eg, [9], [28]), the idea is that overgeneralization errors with a particular verb (e.g., *Daddy laughed the baby) are probabilistically blocked by *any* use of the relevant verb (e.g., *The baby laughed*), and not solely—as for preemption—by uses in a nearly-synonymous construction (e.g., *Daddy made the baby laugh*). In other words, the entrenchment of a verb *in any number of constructions* probabilistically blocks its generalization into constructions in which it has not been attested. In intuitive terms, one can imagine the learning mechanism making a kind of inference from absence (e.g., “Given how frequently I’ve encountered laugh, then if this verb *could* appear in the transitive causative construction, I would surely have heard it by now”). However, entrenchment need not necessarily be framed in terms of deductive reasoning. For example, connectionist networks can show entrenchment type behaviour (eg, [29]) simply because increasing the strength of the connection between an input node representing *laugh* and output nodes representing other constructions (e.g., the intransitive, the periphrastic causative, the single-word imperative) necessarily reduces the strength of the connection between *laugh* and the output node representing the transitive causative construction.

Entrenchment enjoys an important advantage over preemption: Because erroneous uses are probabilistically blocked by *any* use of the relevant verb, regardless of construction, it does not rely on learners encountering particular verbs in very low frequency constructions (e.g., the periphrastic causative and passive). Indeed, for all verb argument structure overgeneralizations, preempting evidence is always, by definition, a subset of entrenching evidence (and often a very small one).

In support of the entrenchment hypothesis, many studies have observed the predicted negative correlation between the acceptability or production probability of errors and overall verb frequency ([9], [12], [15], [16], [17], [18], [19], [22], [29], [30]) In general, these studies have revealed an effect of entrenchment even after controlling for preemption, though a production study of *un-*prefixation [12] observed the opposite pattern, perhaps because—as noted above—preemption is particularly powerful in the case of word-level overgeneralizations of derivational morphology (e.g., *open* preempting *unclose).

In summary, previous findings and theoretical considerations suggest that entrenchment may hold more promise than preemption as a construction general solution to the problem of the retreat from overgeneralization error. At present, however, this conclusion remains tentative for two reasons. The first is that, since measures of entrenchment and preemption (e.g., overall verb frequency and frequency in a particular construction) are invariably highly correlated, the two mechanisms are difficult to distinguish empirically. Indeed, many of the studies cited above did not even attempt to do so. Those that did used regression techniques to partial out the effect of each individual predictor on the dependent variable. However, this solution produces unreliable results when the correlation between the two predictors is very high (e.g., *r* = 0.7 in [18], 2011; *r* = 0.9 in [21]). In the present study, we address this problem by running separate statistical analyses for each predictor.

The second is that, with a single exception, each of these studies has focused on a single construction pair (or “alternation”). This is an important shortcoming, since we already know that—for example—preemption works well for *at least some* types of errors (e.g., morphological overgeneralizations); the issue at stake is the ability of these two statistical learning mechanisms to provide a general solution to the retreat from error across a range of different error types. More generally, as Herb Clark (co-originator of the preemption account) pointed out in a famous paper, one cannot simply assume that one’s “findings generalize beyond the specific sample of language materials…chosen” ([25]: 335). Although Clark focuses mainly on single words, he explicitly notes that the need to demonstrate generalizability beyond the specific items used in the study holds for “words, sentences and other language materials” (p.335), presumably including abstract syntactic constructions. This clearly cannot be done in a study that includes only a single construction pair In the present study we address this problem by using mixed effects models with crossed random effects for participants and items [26], where “items” includes both verbs and syntactic constructions. This strategy allows us to infer that any observed effect of preemption or entrenchment generalizes beyond the particular verbs and constructions included in the present study; clearly a prerequisite for any satisfactory solution to the partial-productivity paradox.

In summary, the present study tested the preemption and entrenchment hypotheses across a range of verb argument structure constructions. The former predicts a negative correlation between the rated acceptability of a particular overgeneralization error and corpus frequency of the relevant verb *in the single most nearly synonymous construction* (see Table 2 for examples). The latter predicts a negative correlation between the rated acceptability of a particular overgeneralization error and the overall frequency of the relevant verb.

## Method

### Ethics statement

The study was approved by the University of Liverpool Ethics Committee. Informed written consent was obtained from adult participants and from parents of participating children (who also gave verbal consent).

### Participants

Participants were 72 children aged 5;2–6;8 (*M* = 5;10), 72 children aged 9;2–10;6 (*M* = 9;11) and 72 adults aged 18;1–22;2 (*M* = 19;1), all reported as showing typical language development. Participants were recruited from schools and a university in the North West of England. The study was approved by the University of Liverpool ethics committee, and informed consent was obtained from all participants. Oral consent was obtained from children, written consent from parents, and from adult participants.

### Verbs and Sentences

Since the design of the study is rather complex, it is probably best understood by consulting the relevant table (Table 2). The description below is intended to outline the logic of the design set out in the table, rather than to constitute a complete free-standing explanation of the design in its own right.

The study used nine different sentence-level verb argument structure constructions grouped into four alternations: Dative (PO-Dative, DO-dative), Causative (Intransitive inchoative, Transitive causative, Periphrastic causative), Locative (Figure locative, Ground locative) and Passive (Active, Passive) Morphological construction and overgeneralizations (eg, *un-*VERB; *unclose) were not included because the relationship between entrenchment and preemption is different for these error types, in that the relevant preempting form generally has a different lexical root (eg, *open* for *unclose) Thus while our conclusions generalize across different types of overgeneralization *of verb argument structure*, they do not generalize across *all* different types of overgeneralization error Indeed, there is some evidence that preemption may be more important for overgeneralization errors at the morphological level ([12], [23])

For each construction (e.g., PO-dative) we selected (a) four verbs that are grammatical in that construction but not the other construction in the alternation (e.g., *carry*, *haul*, *scream*, *shriek*), (b) four verbs that show the opposite profile (e.g., *cost*, *fine*, *refuse*, *deny*) and (c) four verbs that may appear in both constructions (e.g., *give*, *hand*, *show*, *teach*). As these examples indicate, each set of four verbs comprised two higher-frequency verbs and two lower-frequency near synonyms (e.g., *carry+haul*, *scream+shriek*). We then created a sentence for each construction+verb combination. The sentence for each alternation pair and each high/low frequency synonym pair used the same noun phrases (e.g., Bart carried/hauled the box to Lisa; *Bart carried/hauled Lisa the box). For each of these sentence quadruples, we created, using Anime Studio Pro 5.5, a single cartoon animation for which all four sentences would constitute an appropriate description (e.g., Bart lifting a heavy-looking box, carrying it to Lisa and placing it at her feet). The main purpose of the animations was to maintain children’s interest, but they also served to illustrate the intended meaning of the accompanying sentence, and to demonstrate that its veracity was not in doubt (only its grammaticality).

In fact, the design was not quite as balanced as this description implies, due to (a) the inclusion of periphrastic causatives in the causative alternation (b) the non-existence of passive-only verbs (c) the unavailability of close synonyms for non-passivizable verbs and (d) the fact that the active construction in the passive alternation is the same construction as the transitive construction in the causative alternation (although as a non-causative transitive, it is arguably not *exactly* the same, depending on whether or not one posits multiple transitive constructions; see Ambridge &, Lieven, in press, for discussion). Nevertheless, as Table 2 shows, it was still possible to devise sentence stimuli that are consistent with the overall design.

### Rating scale and Procedure

The dependent variable was the acceptability rating for each sentence on a five-point “smiley face” scale (see [15]) The expressions on the faces ranged from sad (leftmost) to neutral (middle) to happy (rightmost). The two leftmost faces were red, the two rightmost faces green and the middle face split with the left-hand half red and the right-hand half green.

The scale can be downloaded from http://journalsplosorg/plosone/article/figure/image?size=large,id=info:doi/101371/journalpone0110009g002 Children indicated their judgments by selecting a red counter (for ungrammatical) or a green counter (for grammatical) and placing it on the relevant face to provide a graded judgment, with responses noted down by the experimenter. Children were told that the red and green counters could be placed on only the red and green faces respectively, except that either counter could be placed on the middle face. Adults marked their ratings directly on the face scale.

The procedure was the same as that used in previous judgment studies of verb argument structure overgeneralization errors (for a more detailed description, see [8], [15]) In brief, children first complete a training session in which they are told that a talking dog (a toy with an internal loudspeaker connected to a laptop computer) is learning to speak English but “because he’s only a dog, sometimes gets it wrong and says things a bit silly”. The child’s task is to help the dog by telling him whether he “said it right, or a bit silly”. The training procedure consists of seven warm-up sentences; the first two completed by the experimenter, the remainder by the child: *The cat drank the milk* (intended rating 5/5), *The dog the ball played with (1/5), *The frog caught the fly* (5/5), *His teeth man the brushed (1/5), *The woman said the man a funny story (2/5), *The girl telephoned her friend the news (3/5) and *The man whispered his friend the joke* (4/5). Note that the final three warm-up sentences are examples of PO→DO dative overgeneralization errors. Although it would have been ideal to avoid using any of the same types of overgeneralization error as in the study proper, this was unavoidable, given the importance of providing children with practice at rating verb argument structure overgeneralization errors. Nevertheless, the warm-up sentences did not use any of the same verbs as test sentences.

After completing the warm-up, children moved on to the main part of the study, which they completed in two sessions on different (usually consecutive) days. Because the total number of trials (*N* = 100) was felt to be too many for young children, each child completed only half of the total number (i.e., 50): One high-low frequency sentence pair for each cell of the design, selected at random on a child-by-child basis (i.e., for any given row in Table 2, any given child completed either the two sentences in the column “Sentence Pair A” or the two sentences in the column “Sentence Pair B”, but never both). Children completed the trials in pseudo-random order with the constraint that neither (a) the same verb (or its high/low frequency equivalent) nor (b) the same construction could occur on consecutive trials.

### Predictor variables

As outlined in the introduction, the *preemption* measure was operationalized as the log frequency of each verb in the single mostly nearly synonymous construction (see Table 2). Entrenchment was operationalized as the log frequency of all uses of that verb (excluding uses as a noun). Frequency counts were taken from SUBTLEX-UK, a 200 million word corpus of subtitles from programmes shown on British television, which has been shown empirically (e.g., via lexical decision tasks [31]) to be more representative of the language heard by speakers of British English than either (a) the British National Corpus (its only serious rival in terms of size) or (b) the equivalent US subtitle corpus.

In order to generate these measures, we obtained counts of each verb in each of our target constructions. (In fact, since for each target construction, only one other construction was designated the preempting construction, such a level of detail was not necessary for the analysis. The aim in obtaining such detailed counts was to create a publically available resource for use in future studies). Since the SUBTLEX-UK corpus is tagged, but not parsed, these counts had to be obtained largely by hand. First we used a program (custom written by the final author) to (a) count the number of uses of each verb and (b) to extract a random sample of 100 sentences of each (or, for verbs with fewer than 100 occurrences, the full set). Two raters then classified each sentence as (a) an instance of one of the constructions shown in Table 2 (PO-dative, DO-dative, Intransitive, Transitive, Periphrastic Causative, Figure-Locative, Ground-Locative, Passive), (b) an “Other” verb use or (c) a non-verb uses (in which case the sentence was replaced with another, and the counts pro-rated accordingly). Missing arguments were allowed, provided that they could be inferred on the basis of the ongoing discourse, and construction classifications were not mutually exclusive. Thus, for example, an utterance such as “John gave a card” would be classified as both a Transitive and a PO-dative. This decision was taken partly on theoretical grounds (i.e., children presumably can and do recover missing arguments from discourse) and partly on practical grounds: insisting that all arguments be explicitly realized would generally have resulted in counts of close to zero for all three-argument constructions (PO/DO-dative, Figure/Ground-locative).

Two coders (Amy Bidgood and Katherine Twomey) each classified 50% of the dataset, and reliability-checked 10% of the data coded by the other. Inter-rater reliability was 87% (Cohen’s Kappa = 0.79, *z* = 16.4, *p*<0.001). Disagreements were resolved by discussion. Raw verb-in-construction counts were pro-rated, on the basis of the overall number of verb uses, in order to yield a final estimate of the frequency of each verb in each construction in the corpus. All raw data are available in S1 Data.

## Results

As the predictions of the entrenchment and preemption hypotheses relate only to ungrammatical sentences, grammatical sentences were excluded from all analyses (though they play an important role as fillers and encourage use of the full scale).

The data were analysed using linear mixed-effects models ([32]) in *R*, with random intercepts for (a) Participant and (b) Verb (*N* = 32; non-alternating verbs only) nested within Sentence Type (i.e., construction: PO-Dative, DO-Dative, Intransitive, Transitive, Periphrastic causative, Figure locative, Ground locative, Passive) In accordance with the recommendations of a recent methods paper [33], all models included by-participant random slopes, always correlated with the intercept, and by-Sentence Type/Verb random slopes, correlated with the intercept, except for a few cases where this yielded convergence failure. Random slopes for Age Group and its interactions were also excluded for this reason. Depending on the analysis, the fixed effect was either the Preemption or the Entrenchment predictor, with some models also including Age Group (5–6, 9–10, Adult) and the relevant interactions. For example, for the first analysis, the model (in R syntax) was

$$\begin{array}{l} Model1=lmer(Rating\sim \;AgeGroup*Preemption\;+\;\left(1+Preemption|Participant\right)\;+\\ \left(1+Preemption|SentenceType/Verb\right),\;data=UngrammaticalSentences) \end{array}$$

Note that because the nesting structure is rather unusual—transitive-only verbs were rated in both intransitive and periphrastic causative sentences, whereas all other verb types were rated in one sentence type only—it was necessary to specify this structure directly in the syntax. *P* values were obtained via the *t* distribution (from the *lmer* function of *lme4*), but we also confirmed that *p* values obtained using a backwards-elimination model-comparison procedure (performed automatically using the *step* function from the *lmerTest* package, eliminating fixed effects only) yielded an identical pattern of results. Indeed, in most cases the *p* values were identical to at least two decimal places (and hence we do not report them separately).

It is also important to note that the present analysis tests the entrenchment and preemption hypotheses across different sentence constructions (i.e., treating construction as a random effect), but does not look for entrenchment and preemption effects across verbs within any given construction. Given that, for each particular construction, only four verbs—and hence four sentences—are ungrammatical, such an analysis would be seriously underpowered, and almost guaranteed to yield Type II errors (i.e., to fail to detect any effect present). Such an analysis strategy is not at all unusual. For example, consider a hypothetical drug trial in which 32 human participants are split across 8 treatment centers, with four participants per center. (analogous to the present situation of 32 verbs nested across 8 constructions). Mixed effects modeling (with treatment center as a random effect) could tell us that the drug is effective, and that this effect generalizes across the 8 treatment centers, but could not tell us whether or not the treatment given in any one center alone was effective. In the same way, the present study can tell us whether entrenchment and preemption effects are observed, and generalize across constructions, but not whether they hold for any particular construction individually.

### Preemption

**Fig 1:**
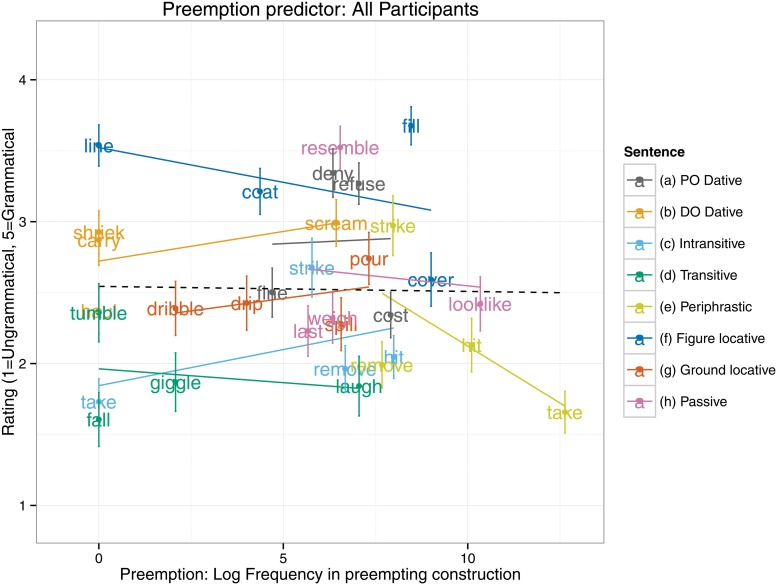
Preemption predictor: All Participants.

The first analysis (see Table 3) was conducted on the combined data for all participants, and hence, in addition to the Preemption predictor, included as fixed effects Age Group and its interactions (with Adult as the reference category). This analysis revealed a main effect of age, such that both 5–6 and 9–10 year olds rated the overgeneralized ungrammatical sentences as more acceptable than did adults. However, the preemption predictor was not associated with any main effects or interactions (*t* <1 in all cases). The null effect for the preemption predictor (collapsing across all age groups) is plotted in Fig 1; it is clear that the line is almost flat. Despite the lack of a significant interaction of Age Group by Preemption, it seemed important to verify that no individual age group showed any suggestion of a preemption effect, by running a separate model for each. These models (see Table 4) revealed no effect of preemption for any group (*t*<1 in all cases). Thus, in summary, the present study failed to find any evidence for preemption either for all participants combined, or for any age group individually.

**Table 3:** Statistical models for all participants combined.

Factor (Preemption model)		Estimate	StdError	df	t value	Pr(>|t|)	Sig
(Intercept)		196	025	780	798	000	***
Age 5 (vs Adults)		108	011	27010	950	000	***
Age 9 (vs Adults)		040	011	26950	350	000	***
Preemption		002	003	1300	052	061	
Age 5 x Preemption		-001	001	288400	-055	058	
Age 9 x Preemption		001	001	288200	083	041	
**Random (Preemption model)**		**Variance**	**StdDev**	**Corr**			
Participant	(Intercept)	018	043				
	Preemption	000	001	100			
Verb x Stype	(Intercept)	010	032				
	Preemption	001	011	-100			
Stype	(Intercept)	027	052				
	Preemption	000	003	-100			
	Residual	134	116				
**Factor (Entrenchment model)**		**Estimate**	**StdError**	**df**	**t value**	**Pr(>|t|)**	**Sig**
(Intercept)		269	035	3940	771	000	***
Age 5 (vs Adults)		047	023	53320	206	004	*
Age 9 (vs Adults)		057	023	53250	249	001	*
Entrenchment		-008	005	3700	-167	010	
**Age 5 x Entrenchment**		**007**	**002**	**293150**	**277**	**001**	**
Age 9 x Entrenchment		-001	002	292710	-054	059	
**Random (Entrenchment model)**		**Variance**	**StdDev**	**Corr**			
Participant	(Intercept)	027	052				
	Entrenchment	000	001	-100			
Verb x Stype	Entrenchment	000	005				
Stype	Entrenchment	000	005				
	Residual	133	115				
**Factor (Preemption model)**		**Estimate**	**StdError**	**df**	**t value**	**Pr(>|t|)**	**Sig**
(Intercept)		196	025	780	798	000	***
Age 5 (vs Adults)		108	011	27010	950	000	***
Age 9 (vs Adults)		040	011	26950	350	000	***
Preemption		002	003	1300	052	061	
Age 5 x Preemption		-001	001	288400	-055	058	
Age 9 x Preemption		001	001	288200	083	041	
**Random (Preemption model)**		**Variance**	**StdDev**	**Corr**			
Participant	(Intercept)	018	043				
	Preemption	000	001	100			
Verb x Stype	(Intercept)	010	032				
	Preemption	001	011	-100			
Stype	(Intercept)	027	052				
	Preemption	000	003	-100			
	Residual	134	116				
**Factor (Entrenchment model)**		**Estimate**	**StdError**	**df**	**t value**	**Pr(>|t|)**	**Sig**
(Intercept)		269	035	3940	771	000	***
Age 5 (vs Adults)		047	023	53320	206	004	*
Age 9 (vs Adults)		057	023	53250	249	001	*
Entrenchment		-008	005	3700	-167	010	
**Age 5 x Entrenchment**		**007**	**002**	**293150**	**277**	**001**	**
Age 9 x Entrenchment		-001	002	292710	-054	059	
**Random (Entrenchment model)**		**Variance**	**StdDev**	**Corr**			
Participant	(Intercept)	027	052				
	Entrenchment	000	001	-100			
Verb x Stype	Entrenchment	000	005				
Stype	Entrenchment	000	005				
	Residual	133	115				

**Table 4:** Statistical models for each age group separately.

Preemption, Age 5–6							
Factor (Final model)		Estimate	StdError	df	t value	Pr(>|t|)	Sig
(Intercept)		312	010	11191	3257	<2e-16	***
Preemption		-001	002	6777	-058	057	
**Random (Final model)**		**Variance**	**StdDev**	**Corr**			
Participant	(Intercept)	019	043				
	Preemption	000	001	100			
Verb x Stype	Preemption	000	003592				
Stype	Preemption	000	0				
	Residual	201	142				
**Entrenchment, Age 5–6**							
**Factor (Final model)**		**Estimate**	**StdError**	**df**	**t value**	**Pr(>|t|)**	**Sig**
(Intercept)		323	029	3918	1125	0000	***
Entrenchment		-002	004	3376	-050	0618	
**Random (Final model)**		**Variance**	**StdDev**	**Corr**			
Participant	(Intercept)	008	027				
	Entrenchment	000	003	100			
Verb x Stype	Entrenchment	000	003				
Verb x Stype1	(Intercept)	000	000				
Stype	Entrenchment	000	002				
Stype1	(Intercept)	000	000				
Residual		194	139				
**Preemption Age 9–10**							
**Factor (Final model)**		**Estimate**	**StdError**	**df**	**t value**	**Pr(>|t|)**	**Sig**
(Intercept)		245	028	793	892	000	***
Preemption		001	004	2112	020	084	
**Random (Final model)**		**Variance**	**StdDev**	**Corr**			
Participant	(Intercept)	020	044				
	Preemption	000	001	100			
Verb x Stype	(Intercept)	032	0567182				
	Preemption	000	005	-026			
Stype	(Intercept)	019	0433776				
	Preemption	000	000	100			
	Residual	116	108				
**Entrenchment Age 9–10**							
**Factor (Final model)**		**Estimate**	**StdError**	**df**	**t value**	**Pr(>|t|)**	**Sig**
(Intercept)		357	051	3100	701	0000	***
**Entrenchment**		**-013**	**006**	**2879**	**-228**	**0031**	*
**Random (Final model)**		**Variance**	**StdDev**	**Corr**			
Participant	(Intercept)	042	065				
	Entrenchment	000	005	-068			
Verb x Stype	Entrenchment	000	000				
Verb x Stype1	(Intercept)	028	053				
Stype	Entrenchment	000	000				
Stype1	(Intercept)	015	039				
Residual		115	107				
**Preemption Adults**							
**Factor (Final model)**		**Estimate**	**StdError**	**df**	**t value**	**Pr(>|t|)**	**Sig**
(Intercept)		207	033	687	626	000	***
Preemption		-001	004	1192	-015	088	
**Random (Final model)**		**Variance**	**StdDev**	**Corr**			
Participant	(Intercept)	017	041				
	Preemption	000	001	-100			
Verb x Stype	(Intercept)	012	035				
	Preemption	000	004	100			
Stype	(Intercept)	053	073				
	Preemption	000	004	-100			
	Residual	068	082				
**Entrenchment Adults**							
**Factor (Final model)**		**Estimate**	**StdError**	**df**	**t value**	**Pr(>|t|)**	**Sig**
(Intercept)		340	051	1223	664	0000	***
**Entrenchment**		**-016**	**004**	**2031**	**-365**	**0002**	**
**Random (Final model)**		**Variance**	**StdDev**	**Corr**			
Participant	(Intercept)	060	078				
	Entrenchment	000	005	-100			
Verb x Stype	(Intercept)	280	167				
Entrenchment		002	013	-100			
Stype	(Intercept)	050	071				
Entrenchment		000	003	-100			
	Residual	067	082				

### Entrenchment

**Fig 2:**
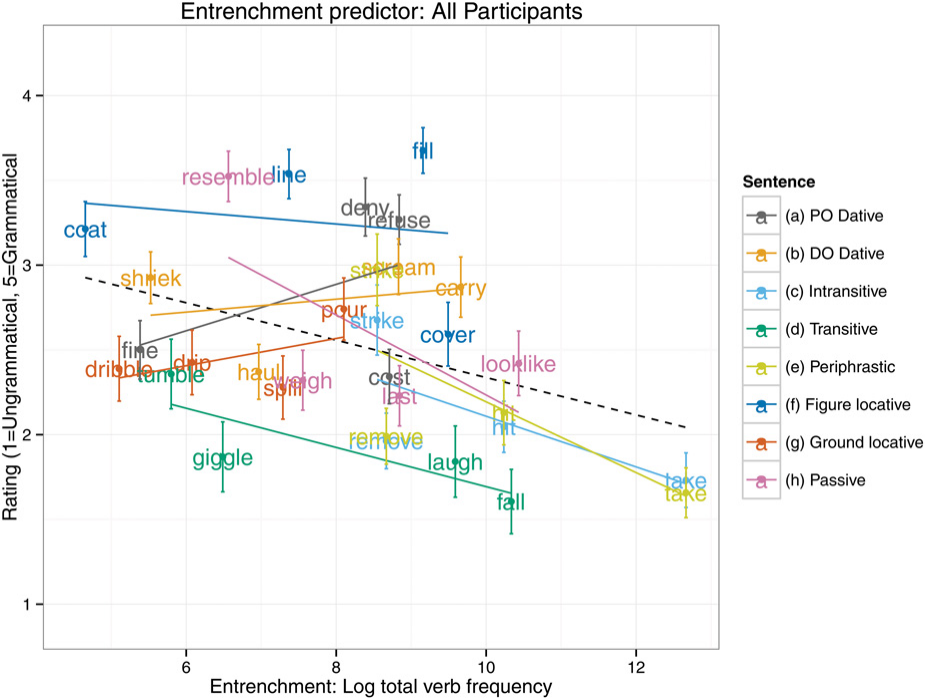
Entrenchment predictor: All Participants.

**Fig 3:**
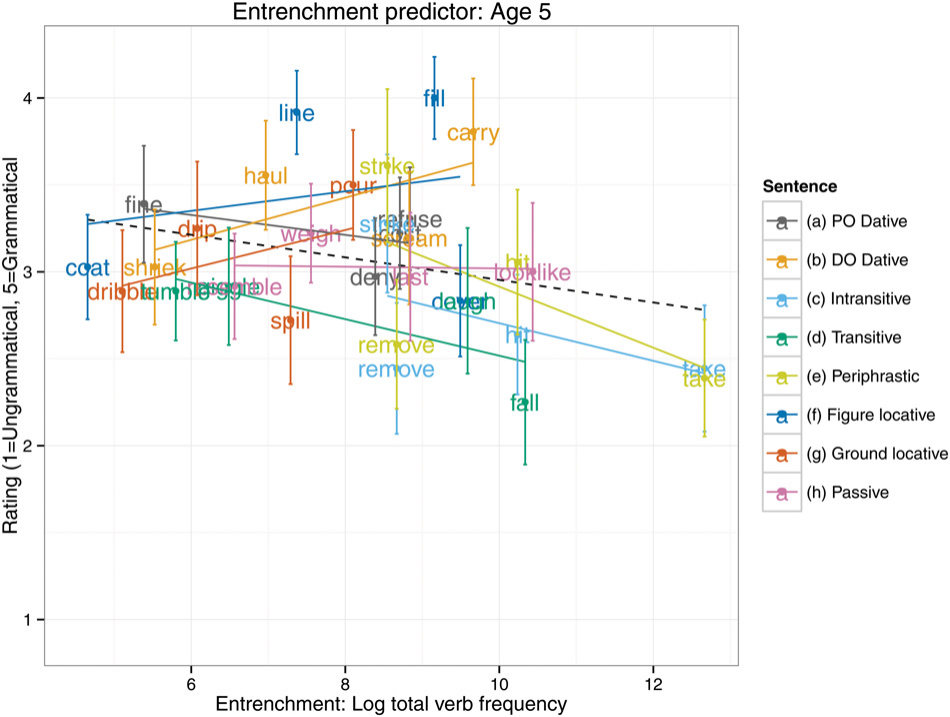
Entrenchment predictor: Age 5–6.

**Fig 4:**
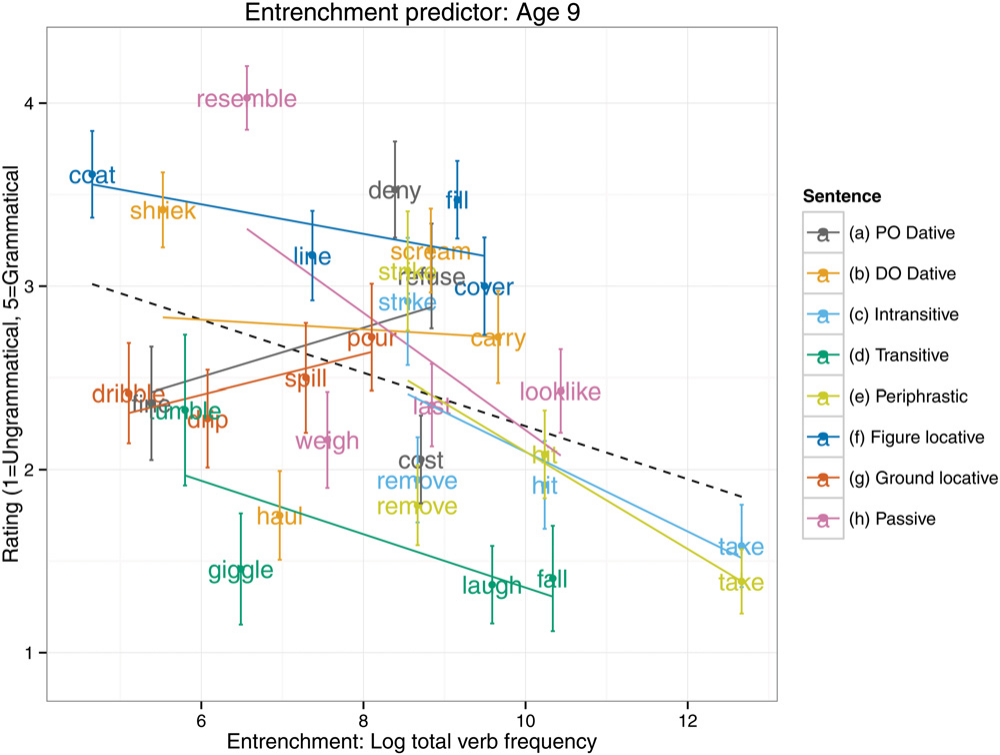
Entrenchment predictor: Age 9–10.

**Fig 5:**
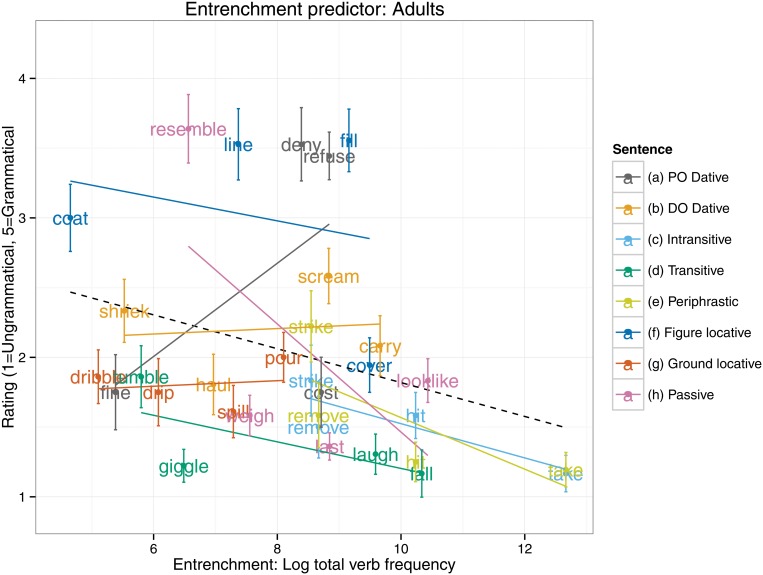
Entrenchment predictor: Adults.

An equivalent set of analyses for the entrenchment predictor (see Table 3) revealed an interaction, such that 5–6 year olds, but not 9–10 year olds, showed a significantly smaller entrenchment effect than did adults. Indeed, the follow up models (see Table 4) revealed that a significant entrenchment effect in the predicted (negative) direction was displayed by the 9–10 year olds (*B* = -0.13, *SE* = 0.06, *t*[28.79] = -2.28, *p* = 0.03) and adults (*B* = -0.16, *SE* = 0.04, *t*[20.31] = -3.65, *p* = 0.002), but not the 5–6 year olds (*t*<1). Presumably the null finding for the 5–6 year olds is the cause of the narrow failure of the main effect of entrenchment to reach significance (*p* = 0.10) in the all-participants analysis (see Table 3 and Fig 2). Plots of the entrenchment predictor for each age group separately (Figs 3–5) show that as overall verb frequency increases, so the rated acceptability of errors decreases (significantly so for the two older groups). Thus, in summary, the present study found an effect for entrenchment for 9–10 year olds and adults, but not 5–6 year olds.

## Discussion

The present study investigated the central question of how children retreat from—or, in many cases, avoid altogether—errors of verb argument structure overgeneralization (e.g., *Daddy giggled the baby). The study was motivated by previous empirical findings and theoretical considerations which raise doubts regarding the feasibility of preemption as a general solution to the problem of the retreat from argument structure overgeneralization, and suggest that entrenchment may constitute a more promising approach.

To investigate this possibility, participants aged 5–6, 9–10 and 18–22 rated the acceptability of overgeneralization errors with a variety of different constructions. No support was found for the prediction of the preemption hypothesis that the greater the frequency of the verb in the single most nearly synonymous construction (for this example, the periphrastic causative; e.g., *Daddy made the baby giggle*), the lower the acceptability of the error. Support was found, however, for the prediction of the entrenchment hypothesis that the greater the frequency of the verb in all constructions (e.g., *The baby laughed*), the lower the acceptability of the error, at least for 9–10 year olds and adults. Although previous studies have investigated the preemption and entrenchment hypotheses, the present study was unique in using statistical models with crossed random effects for participants and items (both verb and construction) in order to investigate the generalizability of these effects. The conclusion, then, is that entrenchment appears to be a robust effect that generalizes across different types of verb argument structure overgeneralization error. (see also [34]) We did not, however, find any evidence that this is the case for preemption.

Before considering this null effect in more detail, it is important to reemphasize that the present study investigated only sentence-level overgeneralizations of verb argument structure. It remains possible, even likely, that preemption is the major retreat mechanism for other types of overgeneralization such as morphological overgeneralizations at the lexical level (e.g., *whisk* and *open* preempt *whisker and *unclose). Indeed, as we saw in the introduction, the preemption account was initially proposed with exactly these types of errors in mind [5] It is the subsequent extension of this account to overgeneralizations of verb argument structure that the findings of the present study call into question.

Returning to the present findings, although it is always difficult to draw conclusions on the basis of a null effect, the lack of a preemption effect does not seem to be straightforwardly attributable to flaws in our experimental design. For example, the study does not seem to be underpowered with regard to the number of participants (72 at each age). Indeed, given that the regression line is almost flat, it does not seem likely that adding even a large number of participants would change the outcome.

Another potential objection is that we failed to select a set of verbs with a sufficient spread along the dimension defined by the preemption predictor (i.e., that there is too little variance in this measure to enable it to predict variance in participants’ judgments). Certainly it is true that, by definition, the spread is smaller for the preemption than the entrenchment predictor. Nevertheless, inspection of Fig 1 reveals that the preemption predictor shows a relatively good spread; and, again, the finding that the predictor did not even approach significance suggests that a preemption effect could not easily be obtained simply by adding more items.

A related objection is that the absolute frequency of the verbs in the relevant preempting constructions was too low for a preemption effect to be observed. However, this is exactly the point: While preemption may work in experimental novel verb studies in which participants are trained on a very large number of exemplars, the present study suggests that, for many familiar verbs, even adults may never encounter sufficient preempting evidence: occurrence in very low frequency constructions such as the passive and periphrastic causative. (Of course, learners hear enough exemplars of these constructions to eventually acquire them, but this does not necessarily mean that they hear each and every relevant verb used in one of these constructions).

In summary, although it is wise to avoid drawing firm conclusions on the basis of a single experimental result, particularly when it is a null effect, the present study at least raises doubt regarding the feasibility of preemption as a general solution to the retreat from sentence-level overgeneralizations of verb argument structure. This raises the question of how to explain the finding that preemption *does* seem to work for certain verb argument structure constructions, particularly the locative constructions [12] (though it is important to remember that the design of the present study did not allow for the investigation of pre-emption or entrenchment effects for any particular construction individually). What, in fact, does it mean to have a learning mechanism that works for some constructions, but not others? Why would children use preemption in only a particular subset of cases to which it would seem to apply?

The answer, we suggest, is that it is a mistake to posit a sharp distinction between preemption and entrenchment, and perhaps even to posit preemption and entrenchment as *mechanisms* rather than *effects* at all. Consider an account under which several different constructions (e.g., active transitive, passive transitive, intransitive, periphrastic causative) compete for the right to express the speaker’s message (eg, DADDY CAUSE [BABY LAUGH]; *Daddy laughed the baby; *The baby was laughed by Daddy; The baby laughed; Daddy made the baby laugh), perhaps even in real time as the sentence is produced word-by-word Assume that the activation of each competitor construction is determined, at least in part, by the frequency with which the verb that the speaker intends to use has occurred in each. An “entrenchment” effect would fall naturally out of this competition process, with no need for any kind of semi-explicit inference from absence. Now, one *could* draw a circle around a particular set of verb uses (for this example, periphrastic causative uses), and label them as “preemption”, but this would seem to add little to the explanation: Both “entrenchment” and “preemption” are just labels for particular *effects* that are outcomes of the construction competition process, rather than *mechanisms*.

Why then—according to previous studies—are preemption effects observed for some constructions but not others? Under the account that we have outlined above, preemption is simply a special subtype of entrenchment: A preemption effect (as opposed to solely entrenchment) will be observed when the “preempting” construction is (a) particularly frequent relative to the error construction and (b) particularly closely synonymous with the error (see [29] for evidence that a connectionist model that implements these factors can yield entrenchment and preemption effects in this way). Presumably these conditions are met for overgeneralizations of PO-dative-only verbs into the DO-dative construction (e.g., *I said no to her* preempts *I said her no*), and indeed for morphological overgeneralization errors (e.g., *ran*, *mice* and *open* preempt *runned; *mouses and *unclose). Presumably, they are not met, however, for a sufficient number of the constructions used in the present study for a significant construction-general preemption effect to be observed.

Essentially the same argument can be made coming from the opposite direction; by positing that entrenchment is a special extension of preemption Suppose that the notion of preemption is broadened such that errors with a particular verb (eg, *Daddy laughed the baby) are probabilistically blocked not only by uses of that verb in the *single* most nearly synonymous construction (eg, *Daddy made the baby laugh*) but by *every* construction that meets some minimum threshold for near synonymy (eg, *The baby laughed*) Under this proposal, preemption and entrenchment are again collapsed into a single construction-competition process

If the account that we have outlined here is along the right lines, then the aim of future research should be not so much to disentangle “preemption” and “entrenchment”, but rather to investigate the factors that determine the outcome of this putative construction-competition process. These factors might include the (a) frequency of the relevant verb and the relevant construction (independently and in co-occurrence), (b) the extent to which the construction is relevant to the speaker’s intended message (and includes a slot for every argument that the speaker intends to express) and (c)—a factor that we have not discussed here—the fit between the verb and the verb slot of the relevant construction in terms of semantics, pragmatics, phonology and any other properties exemplified by this slot (see [12], [13], [17], [18], [19], [20], [21], [22], [29])

In conclusion, whether or not the account set out above is along the right lines, the present study has provided some preliminary evidence against the longstanding claim that preemption is the key mechanism in the retreat from verb argument structure overgeneralization error. Instead, our findings suggest the need for a learning mechanism that is sensitive to overall verb frequency, regardless of construction (whether or not this is framed as “entrenchment”). Although a number of previous studies have investigated both preemption and entrenchment, the novel and particularly important contribution of the present study is its demonstration that only the latter (or whatever takes its place) appears to be robust across a range of different argument structure overgeneralizations. We hope, therefore, that this study will inspire other researchers not only to conduct further experimental investigations into the factors that are important in the retreat from overgeneralization, but also to accept Clark’s [25] challenge of demonstrating that the effects observed—and hence the mechanisms proposed—generalize across different types of errors, and hence hold the promise of a general solution to the paradox of partial productivity.

## Supporting Information

S1 Data Anonymized participant-level data (ie, acceptability ratings on the 5-point scale) and predictor variables (overall and in-construction frequencies of each verb) in CSV format (CSV)
